# Supplementary material for: Placental uptake and metabolism of 25(OH)vitamin D determine its activity within the fetoplacental unit
Source: eLife. 2022 Mar 8;11:e71094. doi: 10.7554/eLife.71094 (PMC8903835; doi:10.7554/eLife.71094)
Supplement: Transparent reporting form [file elife-71094-transrepform1.docx]

***eLife’s* transparent reporting form**

We encourage authors to provide detailed information *within their submission* to facilitate the interpretation and replication of experiments. Authors can upload supporting documentation to indicate the use of appropriate reporting guidelines for health-related research (see [EQUATOR Network](http://www.equator-network.org/%20)), life science research (see the [BioSharing Information Resource](https://biosharing.org/)), or the [ARRIVE guidelines](http://www.plosbiology.org/article/info:doi/10.1371/journal.pbio.1000412) for reporting work involving animal research. Where applicable, authors should refer to any relevant reporting standards documents in this form.

If you have any questions, please consult our Journal Policies and/or contact us: [editorial@elifesciences.org](mailto:editorial@elifesciences.org).

**Sample-size estimation**

- You should state whether an appropriate sample size was computed when the study was being designed
- You should state the statistical method of sample size computation and any required assumptions
- If no explicit power analysis was used, you should describe how you decided what sample (replicate) size (number) to use

Please outline where this information can be found within the submission (e.g., sections or figure legends), or explain why this information doesn’t apply to your submission:

Due to a lack of trial data for these novel studies it was not possible to perform meaningful power calculations during the design of these studies. However, we were able to draw on our experience of studying amino acid and corticosteroid metabolism in the perfused placenta when deciding on the necessary sample size. For the RNA studies, using Southampton women’s survey data our sample size was 102 placentae, which was determined by the available samples. For each of these placentas, five samples selected on a stratified random basis were pooled and powdered in a frozen tissue press (in methods) to ensure representative. The sample numbers can be found in the methods section on page 11 of the manuscript. The exact sample numbers for each fragment experiment are in the legend of Figure 3 as these are defined as a range in the methods. The sample numbers for the precise comparisons (based on anthropometric data availability) in the SWS data set are presented in Table 1.

**Replicates**

- You should report how often each experiment was performed
- You should include a definition of biological versus technical replication
- The data obtained should be provided and sufficient information should be provided to indicate the number of independent biological and/or technical replicates
- If you encountered any outliers, you should describe how these were handled
- Criteria for exclusion/inclusion of data should be clearly stated
- High-throughput sequence data should be uploaded before submission, with a private link for reviewers provided (these are available from both GEO and ArrayExpress)

Please outline where this information can be found within the submission (e.g., sections or figure legends), or explain why this information doesn’t apply to your submission:

Placental perfusions each require whole placenta so no technical replicates within experiment.

Villous fragment culture and cytotrophoblast culture 3 replicate fragments per treatment for each placenta = matched samples (in methods).

qPCR samples in triplicate (in methods).

Sequencing data uploaded and described in data availability statement.

RNA sequencing data, ChIP-seq data and methylation array data that support the findings of this study have been deposited in the Gene Expression Omnibus (GEO) with the accession code GSE167431.

All protein mass spectrometry data have been deposited to the ProteomeXchange Consortium via the Proteomics Identifications Archive with the data set identifier PXD011443.

All data were included; missing values are due to data not being available/measureable.

**Statistical reporting**

- Statistical analysis methods should be described and justified
- Raw data should be presented in figures whenever informative to do so (typically when N per group is less than 10)
- For each experiment, you should identify the statistical tests used, exact values of N, definitions of center, methods of multiple test correction, and dispersion and precision measures (e.g., mean, median, SD, SEM, confidence intervals; and, for the major substantive results, a measure of effect size (e.g., Pearson's r, Cohen's d)
- Report exact p-values wherever possible alongside the summary statistics and 95% confidence intervals. These should be reported for all key questions and not only when the p-value is less than 0.05.

Please outline where this information can be found within the submission (e.g., sections or figure legends), or explain why this information doesn’t apply to your submission:

(For large datasets, or papers with a very large number of statistical tests, you may upload a single table file with tests, Ns, etc., with reference to sections in the manuscript.)

Statistical analysis is described in the methods for each experiment along with n numbers (the exact n numbers for the fragment experiments are in the figure legend as these are defined as a range in the methods). The n numbers for the precise comparisons in the SWS data set are presented in a table.

Precision measures and p values are in the figure legends.

Main results are in the results and discussion section.

**Group allocation**

- Indicate how samples were allocated into experimental groups (in the case of clinical studies, please specify allocation to treatment method); if randomization was used, please also state if restricted randomization was applied
- Indicate if masking was used during group allocation, data collection and/or data analysis

Please outline where this information can be found within the submission (e.g., sections or figure legends), or explain why this information doesn’t apply to your submission:

For vitamin D treatment experiments, each placenta had matched samples in the treatment and control group (randomised).

For perfusion experiments all placentas were treated the same and the samples generated were measured for metabolomics in a blinded manner.

The end stage measurement and analysis for proteomics, gene expression, DNA methylation was carried out blinded.

**Additional data files (“source data”)**

- We encourage you to upload relevant additional data files, such as numerical data that are represented as a graph in a figure, or as a summary table
- Where provided, these should be in the most useful format, and they can be uploaded as “Source data” files linked to a main figure or table
- Include model definition files including the full list of parameters used
- Include code used for data analysis (e.g., R, MatLab)
- Avoid stating that data files are “available upon request”

Please indicate the figures or tables for which source data files have been provided:

RNA sequencing data and methylation array data that support the findings of this study are included as supplementary files.

The placental perfusion metabolism data is uploaded as a source file.

The qPCR data plus analysis code is uploaded as a source file.
